# The determination of the biological function of bacterial pink pigment and *Fusarium chlamydosporum* on alfalfa (*Medicago sativa* L.)

**DOI:** 10.3389/fmicb.2023.1285961

**Published:** 2023-10-19

**Authors:** Rong Huang, Hong Zhang, Haiyan Chen, Linxin He, Xiaoni Liu, Zhenfen Zhang

**Affiliations:** Key Laboratory of Grassland Ecosystem, Ministry of Education, Sino-U.S. Centers for Grazing Land Ecosystem Sustainability, Ministry of Science and Technology, Pratacultural College, Gansu Agricultural University, Lanzhou, China

**Keywords:** *Erwinia persicina*, bacterial pink pigment, *Fusarium chlamydosporum*, alfalfa, plant growth and physiology

## Abstract

Bacterial pigment is one of the secondary metabolites produced by bacteria and has functions that are yet to be understood in relation to soil-borne pathogenic fungi and plants in mutualistic processes. The study evaluates the growth, photosynthetic, and physiological characteristics of alfalfa after interacting with different concentrations of Cp2 pink pigment and *Fusarium chlamydosporum*. The findings showed that Cp2 pink pigment has the ability to inhibit the growth of alfalfa, with the inhibition ratio gradually increasing with rising concentration. *F. chlamydosporum* inhibited the growth of alfalfa, which reduced the photosynthetic physiological response and elevated antioxidant enzymes, which are typically manifested by yellowing leaves and shortened roots. Under the combined effect of Cp2 pink pigment and *F. chlamydosporum*, increasing concentrations of Cp2 pink pigment intensified the symptoms in alfalfa and led to more pronounced growth and physiological response. This indicates that the Cp2 pink pigment is one of the potential virulence factors secreted by the *Erwinia persicina* strain Cp2, which plays an inhibitory role in the interactions between *F. chlamydosporum* and alfalfa, and also has the potential to be developed into a plant immunomodulator agent.

## Introduction

Alfalfa (*Medicago sativa* L.) is rich in valuable nutrients, including essential proteins, minerals, amino acids, vitamins, carotene, dietary fiber, and flavonoids, as found in perennial herbaceous plants (Chen et al., 2020; Hadidi et al., 2022), which belongs to the *Medicago* of Fabaceae family, and it is known as “the king of forages” (Nemchinov et al., 2017; Dhakal et al., 2020).

Alfalfa is a powerful and high-quality forage that supports the restructuring of China's livestock production in the twenty first century (Rajat and Manisha, 1997; Dhakal et al., 2020). However, as the area of alfalfa cultivation and the number of planting years grow year by year, alfalfa disease problems also increase.

According to the statistics, diseases are significant threats to alfalfa and can reduce alfalfa yields by up to 20% (Wang et al., 2020a,b).

For example, among the main *Fusarium* pathogens causing root rot are *Fusarium* oxysporum, *Fusarium solani*, and *Fusarium chlamydosporum* species complex (Cong et al., 2016). *Fusarium chlamydosporum is* the main pathogen of alfalfa root rot, which is one of the worldwide epidemic pathogens causing alfalfa-producing areas and the main pathogen of alfalfa root rot in Northwest China (Yinghua et al., 2019; Cao et al., 2020; Zhao et al., 2021).

Plant pathogenic bacteria can frequently occupy the surface or tissue of plants, leading to symptoms such as plant wilt, tissue decay, and hormone imbalance (Strange and Scott, 2005). The bioactive compounds that they secrete significantly impact the performance and survival of other organisms (Demain and Sanchez, 2009). Bacterial pigment, which is one of the bioactive secondary metabolites produced by bacteria, exists in nature (Hibbing et al., 2010). Based on their chemical structure, bacterial pigments can be classified into five categories: carotenoids, anthocyanins, polyketide pigments, pyrrole pigments, and others (Mumtaz et al., 2019). The pigment has the ability to bind to numerous biological targets and was found to possess anticancer, antiproliferative, antimicrobial, immunosuppressive, and biodegradable properties (Alem et al., 2022; Brahma and Dutta, 2022; Sudhakar et al., 2022; Yu et al., 2022).

Research on bacterial pigment has been on the rise due to its extensive application value and characteristics (Adhikari and Pandey, 2021). For instance, *Pseudomonas aeruginosa* produces pyocyanin, which serves as a crucial virulence factor and can improve the competitiveness of strains in the environment (Hall et al., 2016), and the deletion mutant of pyocyanin had less significantly aggressive and comparable virulence to the wild-type strain (Lau et al., 2004). According to reports, *Erwinia persicina* strain Cp2 has been identified as the cause of bacterial bud wilt disease in alfalfa, which primarily manifests as symptoms such as water loss, yellowing, and eventually browning, accompanied by a pink solution appearance (Zhang, 2013). He suspected that the pink solution was the bacterial pigment (Cp2 pink pigment). Afterward, Zhang discovered that Cp2 pink pigment displays effective inhibitory effects on various plant pathogenic fungi, including *Alternaria solani, Sclerotinia sclerotiorum, Rhizoctonia solani*, and *F. chlamydosporum*, within 6 days after inoculation (Zhang et al., 2022), and the stability of Cp2 pink pigment to different pH, ionic strength, and temperature conditions was evaluated. However, the functions of Cp2 pink pigment concerning soil-borne pathogenic fungi and their mutualistic interactions with plants remain unclear. Based on the above background, the present study focuses on the antifungal activity of *E. persicina* strain Cp2 pink pigment (Zhang et al., 2022). The second major goal is to explore the Cp2 pink pigment interactions between plants and soil-borne pathogenic fungi, which lays the basis for further clarification of the virulence effects and bactericidal and bacteriostatic activity of Cp2 pink pigment and provides valuable reference information to reveal the mechanism of the pathogenicity of Cp2, as well as a solution for the effective development of plant immunomodulator agents.

## Materials and methods

### Alfalfa, bacteria, and fungi

The seeds of the alfalfa (*Medicago sativa*) cultivar “Juneng 551” were provided by the Sino-U.S. Centers for Grazing Land Ecosystem Sustainability. *E. persicina* strain Cp2 (ID: 756944) was isolated from the internal tissue of alfalfa seeds and stored at −80°C (Zhang, 2013). The *F. chlamydosporum* (ID: 17735672) was obtained from the Laboratory of Plant Pathology, College of Plant Protection, Gansu Agricultural University, Lanzhou, China. Bacterial strains were cultured in nutrient agar media (NA: 3 g beef extract, 10 g peptone, 5 g NaCl, 18 g agar) at 28°C. Fungal culture was grown in potato dextrose agar media (PDA: 200 g potato, 20 g glucose, and 18 g agar) at 25°C and stored at −80°C.

### Pink pigment solution

Cp2 pink pigment was extracted based on the methods used by Zhang et al. (2022). For the crude extraction and purification of the pink pigment, we used organic solvent extraction methods. We collected bacteria on a culture plate, scraped a sample of 1.0 g, and placed it in a 10-mL centrifuge tube. Then, we added 5 mL of 75% alcohol by volume, respectively, to the centrifuge tube with bacteria and soaked it for 1 h. Following the centrifugation at 12,000 rpm for 10 min, the supernatant was collected. Chloroform was added with constant stirring. When pigment precipitated from the upper layer, it was transferred to a separatory funnel and allowed to stand for 2 h until the mixture was divided into three layers. The pigment solution was obtained from the uppermost layer. The produce was then dried at 40 °C to obtain the pure pigment. Finally, it was redissolved in sterile water to obtain a Cp2 pink pigment solution.

The concentration of Cp2 pink pigment was quantified from a standard curve: y = 0.0302 x+1.435 (R^2^ = 0.9994).

Next,1.35 g of Cp2 pink pigment was weighed and dissolved in sterile water to obtain the initial Cp2 pink pigment solution (OD_340_ ≈ 1.582, 4.93 mg/mL). On the basis of pre-experimentation, the Cp2 pink pigment concentration gradient progressive increase induction method was applied, and the Cp2 pink pigment was at a concentration of 0, 0.099, 0.149, 0.198, 0.248, and 0.297 mg·mL^−1^.

### Fungal solution

*F. chlamydosporum* strain was cultured on PDA at 25 °C for 7 d. After incubation in 5 mL of 0.9% sterile sodium chloride solution containing 0.05% polysorbate 80 (Tween 80), the spores were scraped off. Then, the spore suspension was aspirated into a sterile test tube and adjusted with sterile water to an OD of 1.0, with a fungal density of ~10^9^ CFU/mL.

### Experimental design

#### Test equipment

**Figure 1:**
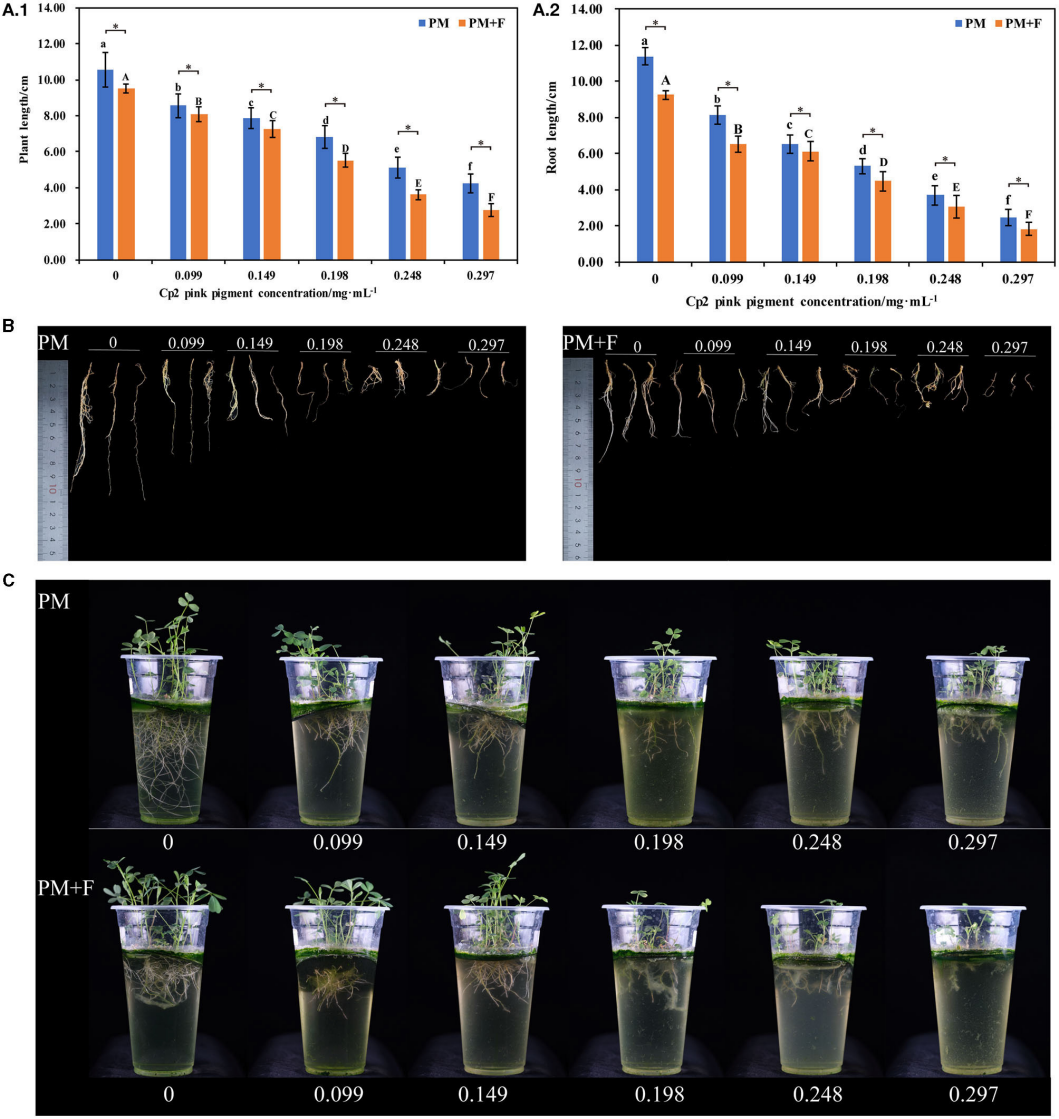
Alfalfa phenotypes and growth indicators at 21 days after inoculation with Cp2 pink pigment and *F. chlamydosporum*. **(A.1)** Comparison of plant length between PM and PM + F groups. **(A.2)** Comparison of root length between PM and PM + F. **(B)** Root phenotypic comparison between PM and PM + F groups. **(C)** Phenotypic comparison between PM and PM + F groups. Bars superscripted by different lowercase letters indicate significant differences in PM (*P* < 0.05), mean ± SE (one-way ANOVA test, *n* = 8); bars superscripted by different capital letters indicate significant differences in PM+ F (*P* < 0.05); *P < 0.05 (*t*-test, *n* = 8); no * indicate no significant difference (*P* > 0.05); the same below.

Transparent polyethylene growth plastic cups (according to the Chinese Utility Patent with minor changes, patent number: ZL-2020-2-1672986.4) were used for plant-growth experiments. The growth plastic cup has three characteristics: first, after 350 mL of solution is loaded into the cup, the liquid surface contacts the undersurface of the germination bed (the germination bed is collected in two layers: the top layer (diameter 7.0 cm) and the sub-top layer (diameter 7.5 cm). The mold is shown in Figure 1C, which ensures that the seeds absorb water normally. Second, the growth cup wrapped in tin foil safeguards the roots of the alfalfa seedling and rhizosphere microorganisms from light exposure, thus ensuring a dynamic balance of the microbial ecosystem in the root. Moreover, you can always keep an eye on the growth status of alfalfa through the transparent growth plastic cups.

### Test method

Prior to starting the plant-growth experiment, the seeds were first sorted and selected to discard those with abnormal or damageable aspects. We used 25 seeds per replicate. For each treatment, the germination bed was placed in growth cups, and then 350 mL of Hoagland's solution was added to the cups. Alfalfa seeds were placed equidistant (seed spacing ≈ 1 cm) on the germination bed of the growth cups (25 seeds/cup) with tweezers.

After inoculation, the cups were then wrapped in foil and placed in a growth chamber at 23°C for daytime temperature and 20°C for nighttime temperature, 45% humidity, and a 16-h day and 8-h night photoperiod. After 8 days, the corresponding concentration gradient of Cp2 pink pigment and *F. chlamydosporum* suspension was added to ensure thorough mixing for plant growth.

The seeds germinated for 8 days. For all treatments, the growth cups were replenished with Hoagland's solution up to the liquid surface. Afterward, the essential volume of Cp2 pink pigment solution at a concentration corresponding to the desired final concentration was added to the growth cups. Finally, we randomly selected half of them for the *F. chlamydosporum* inoculation assay, and 10 mL of the suspension was added to the growth cups.

In this study, Hoagland's solutions supplemented with different volumes of corresponding concentrations of Cp2 pink pigment solution were used as the treatment group, and the group was denoted as the PM group. The same volume of Cp2 pink pigment solution supplemented with 10 mL of fungal suspension was used as another treatment group. They were labeled as the PM + F group. In each of the two treatment groups, only the control was supplemented with only Hoagland's solutions and 0 mg·mL^−1^ Cp2 pink pigment. The control was referred to as the positive control and denoted as CK1. The negative control, denoted as CK2, was supplemented with only Hoagland's solutions and 0 mL of *F. chlamydosporum* (Table 1). A total of 12 treatments were set up, with eight replicates per treatment for a total of 96 plots. During growth, Hoagland's solutions were replenished every 2 days. The liquid surface contacts the undersurface of the germination bed.

**Table 1:** Experimental design.

**Groups**	**Control**			**Treatments**		
PM	0 mg·mL^−1^ Cp2 pink pigment (CK1)	0.099 mg·mL^−1^ Cp2 pink pigment	0.149 mg·mL^−1^ Cp2 pink pigment	0.198 mg·mL^−1^ Cp2 pink pigment	0.248 mg·mL^−1^ Cp2 pink pigment	0.297 mg·mL^−1^ Cp2 pink pigment
PM + F	0 mL *F.chlamydosporum* (CK2)	0.099 mg·mL^−1^ Cp2 pink pigment + 10 mL *F.chlamydosporum*	0.149 mg·mL^−1^ Cp2 pink pigment + 10 mL *F.chlamydosporum*	0.198 mg·mL^−1^ Cp2 pink pigment + 10 mL *F.chlamydosporum* t	0.248 mg·mL^−1^ Cp2 pink pigment + 10 mL *F.chlamydosporum*	0.297 mg·mL^−1^ Cp2 pink pigment + 10 mL *F.chlamydosporum*

### Measurement index and method

#### Measurement of growth-related indicators

After 21 days of seed germination, basic growth indicators, including plant length (PL), root length (RL), aboveground fresh weight (AFW), below-ground fresh weight (BFW), aboveground dry weight (ADW), below-ground dry weight (BDW), were measured (Shao et al., 2016). The Expression 12000XL root system scanning analyzer (Seiko Epson Corporation, Suwa, Nagano, Japan) was used to scan roots, and the morphological indexes (total root area (RA), total root volume (RV), average root diameter (RD), and number of roots (NR) were analyzed using WinRHIZO Basic 2013.

Leaf chlorophyll content was determined using ethanol solvents according to literature with slight modifications (Lichtenthaler, 1987). Briefly, leaf samples (0.1 g) of the PM and PM+F groups were soaked in 95% alcohol in a 5-mL tube. Then, each tube was wrapped in tin foil and placed at room temperature for 24 h. Absorbance readings at 470 nm, 665 nm, and 649 nm for the collected supernatants were used to estimate the contents of the total chlorophyll content (ChlT).

### Determination of photosynthetic parameters of leaves

Photosynthetic parameters of the third apical leaf of plants in the PM and PM + F groups were measured using a portable photosynthesis system (GFS-3000, Heinz Walz, Effeltrich, Germany). Transpiration rate (Tr), stomatal conductance (Gs), net photosynthesis rate (Pn), and intercellular CO_2_ (Ci) were determined at 400 μmol/m^2^/s CO_2_ and 1400 μmol/m^2^/s photosynthesis photon flux density (PPFD). Measurements were performed in the mornings between 9:00 a.m. and 11:00 a.m. on day 21. The photosynthetic parameters (Tr, Gs, Pn, and Ci) were calculated according to Gao et al. (2017).

### SOD, CAT, DHA, MDA, H_2_O_2_, and OFR

Alfalfa seedlings in the PM and PM+F groups were harvested at day 21 and used to determine activities of physiological indicators, including superoxide dismutase (SOD), catalase (CAT), dehydroascorbate (DHA), malondialdehyde (MDA), hydrogen peroxide (H_2_O_2_), and superoxide anion (OFR). The six indicators were determined according to the commercial kits (Suzhou Michy Biomedical Technology Co., Ltd.). Briefly, samples (0.2 g) were homogenized in extraction buffer, centrifuged at 12,000 rpm for 10 min at 4°C, and the resulting supernatants were collected to determine enzyme activities using the corresponding assay kits. The activities of SOD were tested using an ELISA kit (M0102), the activities of CAT were tested using an ELISA kit (M0103), the activities of DHA were tested using an ELISA kit (M0127), the activities of MDA were tested using ELISA kit (M0106), the activities of H_2_O_2_ were tested using an ELISA kit (M0107), the activities of OFR were tested using an ELISA kit OFR(M0114).

### Statistical analysis

The data were collated and graphed using Excel 2019 and Origin 2022. The data were expressed as the mean ± the standard error of the mean from independent replicates and analyzed by SPSS 26.0. The heatmap was carried out using the Chiplot platform (https://www.chiplot.online/).

## Results

### Effect of *F. chlamydosporum* on the plant and root length of alfalfa under different Cp2 pink pigment

With the increase in the concentration of Cp2 pink pigment, the inhibition ratio gradually increased (Figure 1). The stem and leaves gradually became shorter, along with the roots. Concurrently, the lateral roots decreased in number and began to decay over time (Figures 1B, C).

The inhibitory effect of different concentrations of Cp2 pink pigment in the PM + F group on the growth of alfalfa is similar to that in the PM group (Figure 1). As a result of the infestation of *F. chlamydosporum*, the inhibitory effect of the PM + F group on the growth of alfalfa was notably superior to that in the PM group. In addition, we can see in the PM+F group for the 0.099, 0.149, 0.198, 0.248, and 0.297 mg·mL^−1^ treatments that the increase in the concentration of Cp2 pink pigment resulted in a gradual decrease in root tissue and root flocculent attachment growth plastic cups (Figure 1C). To summarize, Cp2 pink pigment and *F. chlamydosporum* can inhibit the growth of alfalfa. Additionally, Cp2 pink pigment expedites the inhibition of *F. chlamydosporum* on the growth of alfalfa.

### Effect of *F. chlamydosporum* on root morphological indexes of alfalfa under different Cp2 pink pigment

**Figure 2:**
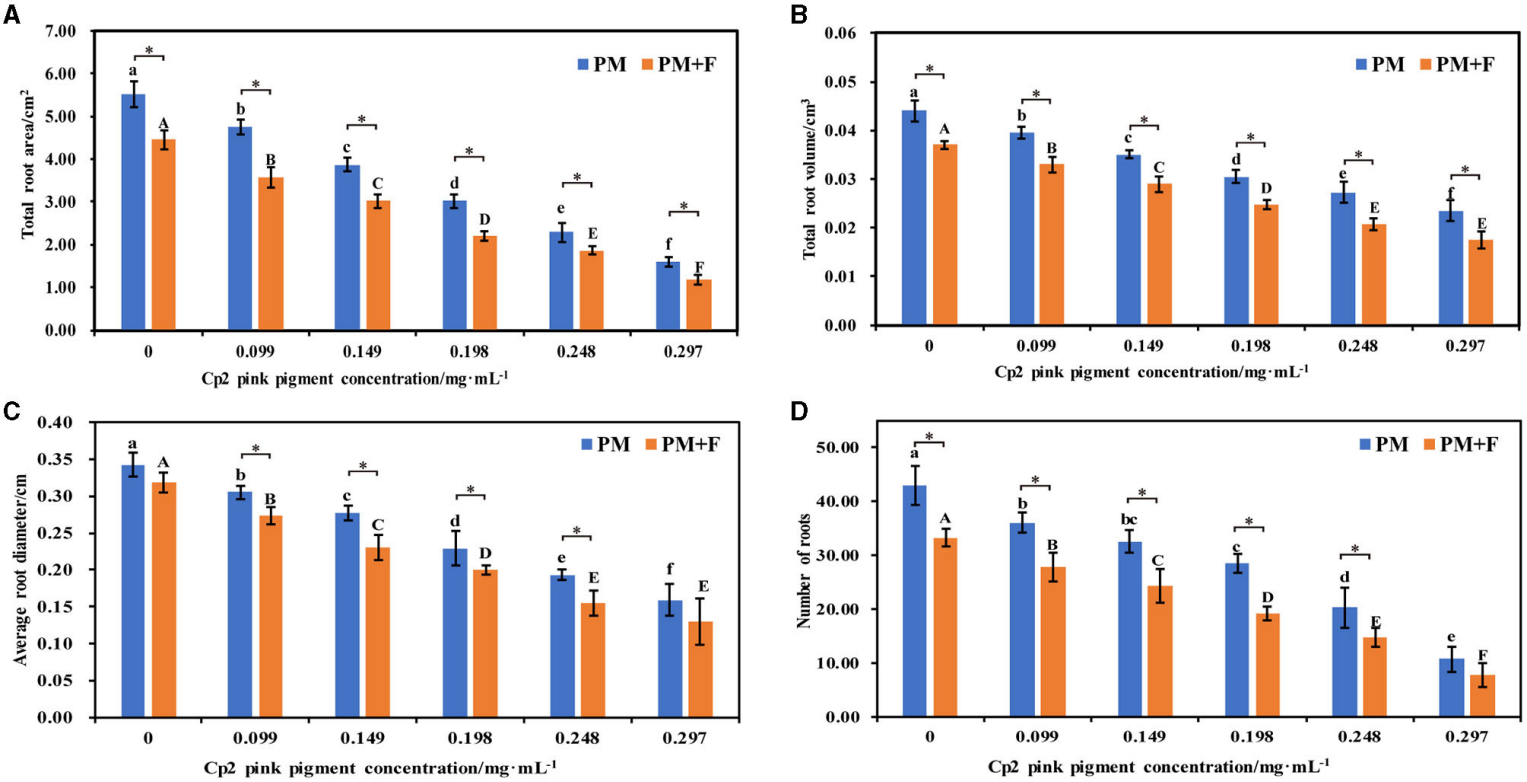
Effects of different concentrations of Cp2 pink pigment and *F. chlamydosporum* on the morphological indexes of alfalfa. **(A)** Total root area (RA). **(B)** Total root volume (RV). **(C)** Root diameter (RD). **(D)** Numbers of roots (NR). *P < 0.05, (*t*-test, *n* = 8); no * indicate no significant difference (*P* > 0.05).

Since the root is the main organ responsible for water and mineral nutrient uptake, we characterized the morphological characteristics of the roots in the PM and PM+F groups. The total root area, total root volume, average root diameter, and number of roots of alfalfa treated with the PM + F group were lower than those in the PM group, and this difference was significant as the concentration of *F. chlamydosporum* suspension increased (*P* < 0.05) (Figures 2A–D). When compared with the control group (CK2), root morphological indexes of alfalfa inoculated with the PM group were lower than the CK2 (Figure 2). Moreover, these parameters decreased gradually with an increase in Cp2 pink pigment concentration. When the concentration of Cp2 pink pigment was between 0.099 and 0.248 mg·mL^−1^, the PM + F group showed significantly lower values for root morphological indexes than the PM group (Figure 2). In summary, the roots became shorter and thicker during the interaction between *F. chlamydosporum*, Cp2 pink pigment, and alfalfa, negatively impacting water and nutrient absorption in the alfalfa roots.

### Effect of *F. chlamydosporum* on the fresh and dry weight of alfalfa under different Cp2 pink pigment

**Figure 3:**
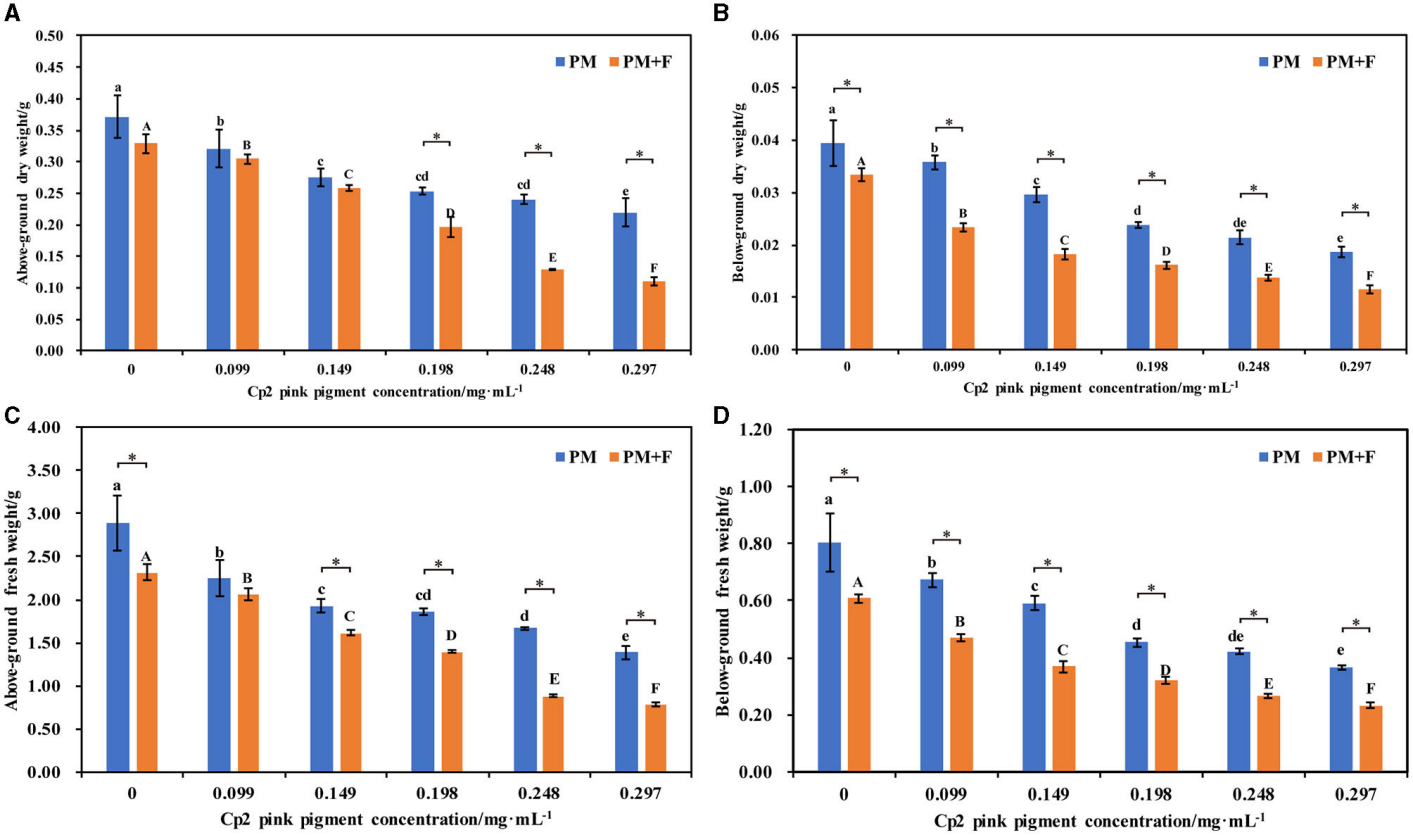
Effects of different concentrations of Cp2 pink pigment and *F. chlamydosporum* on the fresh and dry weight of alfalfa. **(A)** Aboveground dry weight (ADW). **(B)** Below-ground dry weight (BDW). **(C)** Aboveground fresh weight (AFW). **(D)** Below-ground fresh weight (BFW). *P < 0.05, (*t*-test, *n* = 8); no * indicate no significant difference (*P* > 0.05).

Compared with the control (CK1), the aboveground dry weight, below-ground dry weight, aboveground fresh weight, and below-ground fresh weight of alfalfa were significantly worse after inoculation with Cp2 pink pigment. With an increase in the concentration of Cp2 pink pigment, the inhibition ratio gradually increased (Figures 3A–D). They show that Cp2 pink pigment significantly reduces the fresh and dry weights of alfalfa and inhibits their growth. Compared with the control (CK2), the aboveground fresh weight, aboveground dry weight, below-ground fresh weight, and below-ground dry weight of alfalfa were significantly worse after inoculation with the PM+F group, and the inhibition ratio gradually increased with increasing fungal suspension concentration (*P* < 0.05) (Figures 3A–D). When the concentration was higher than 0.149 mg·mL^−1^, the fresh w dry weight of alfalfa in the PM+F group was significantly lower than that in the PM group. From the fresh-dry weight ratio, it was concluded that the water content of alfalfa plants was worse in the PM+F and PM groups compared with the control. Hence, these findings indicate that fungus strain *F. chlamydosporum* and Cp2 pink pigment exhibit a general inhibitory effect on the growth of alfalfa plants, and Cp2 pink pigment accelerates the inhibitory effect on alfalfa.

### Effect of *F. chlamydosporum* on the ChlT of alfalfa under different Cp2 pink pigment

**Figure 4:**
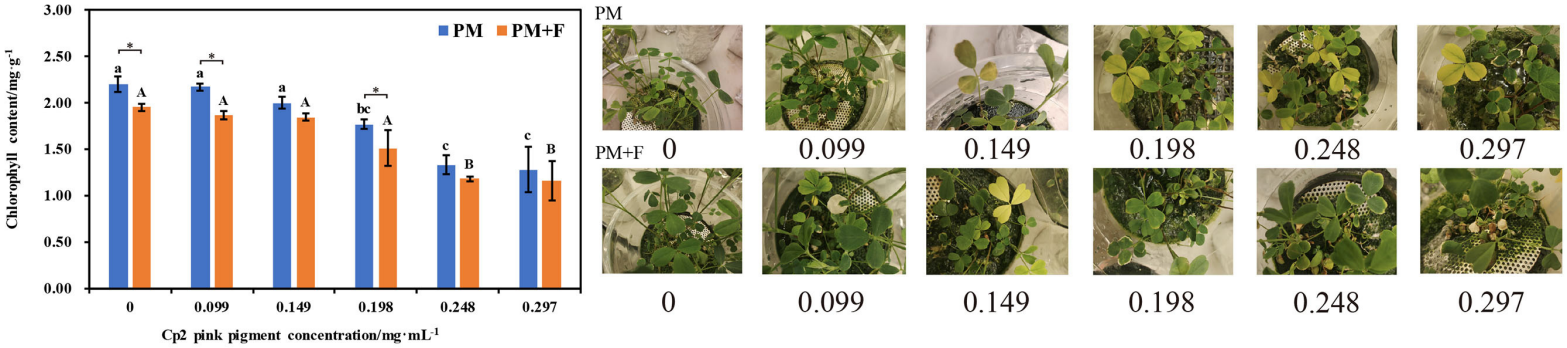
Symptoms on alfalfa leaves, including effects of different concentrations of Cp2 pink pigment and *F. chlamydosporum* on the Chlorophyll content of alfalfa. *P < 0.05, (*t*-test, *n* = 8); no * indicate no significant difference (*P* > 0.05).

From the growth phenotype of the leaves, as the concentration of Cp2 pink pigment increased in the PM group, the inhibitory effect of varying concentrations of Cp2 pink pigment on the growth of alfalfa became more pronounced, exhibiting symptoms such as overall health deterioration, partial yellowing, or even drying out (Figure 4). The inhibitory effect of Cp2 pink pigment in the PM + F group on the growth of alfalfa was comparable to that of the PM group at different concentrations. The main distinction between the two groups lies in the fact that the leaves of alfalfa in the PM group are predominantly yellow, whereas those in the PM + F group are more parched (Figure 4). After 21 days of growth, the ChlT content of alfalfa leaves was determined under each treatment regimen. There was a visible downward trend in the overall ChlT content of alfalfa and an obvious downward trend when concentrations of Cp2 pink pigment in the PM group increased (Figure 4). When the concentration of Cp2 pink pigment was > 0.198 mg·mL^−1^, all the treatments showed a significant effect on ChlT content (*P* < 0.05). The trend of ChlT content in the PM + F group was entirely consistent with that of the PM group. Furthermore, the ChlT content in alfalfa leaves within the PM + F group was inferior to that in the PM group, exhibiting a notable contrast between the two groups when the pigment concentration was at 0.297 mg·mL^−1^.

### Effect of *F. chlamydosporum* on the photosynthetic physiology indexes of alfalfa under different Cp2 pink pigment

**Figure 5:**
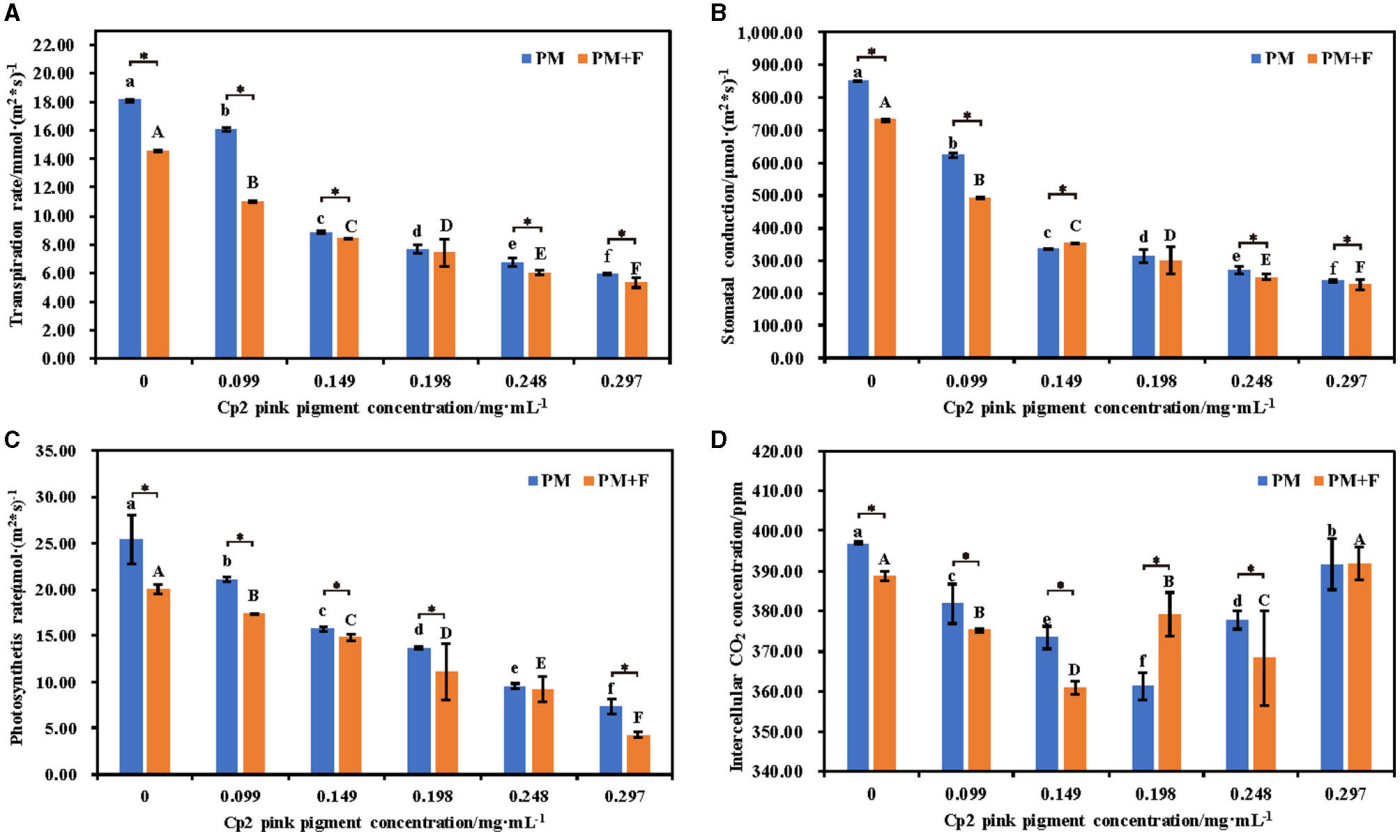
Effects of different concentrations of Cp2 pink pigment and *F. chlamydosporum* on the root morphological indexes of alfalfa. **(A)** Transpiration rate (Tr). **(B)** Stomatal conductance (Gs). **(C)** Photosynthesis rate (Pr). **(D)** Intercellular CO_2_ (Ci). *P < 0.05, (*t*-test, *n* = 8); no * indicate no significant difference (*P* > 0.05).

Measurement of the photosynthetic physiology of leaves revealed that with the increase in Cp2 pink pigment concentration, the Pn, Gs, and Tr of alfalfa experienced a decline in the PM and PM + F groups. As the concentration of Cp2 pink pigment increased, the Pn, Gs, and Tr of alfalfa experienced a downward trend, and the trend became significantly pronounced with the increase in concentration (*P* < 0.05) (Figures 5A–D). Additionally, the three photosynthetic indexes of alfalfa leaves in the PM + F group were inferior to those in the PM group, exhibiting significant distinctions between the two groups when the Cp2 pink pigment concentration was at 0.099 and 0.149 mg·mL^−1^(*P* < 0.05) (Figure 5). The Ci concentration differs from the other three photosynthetic indicators. As the Cp2 pink pigment concentration increased, the Ci concentration in the PM and PM + F groups generally rose first and then fell (Figure 5D). When the Cp2 pink pigment concentration was 0.198 mg·mL^−1^, it signified a major turning point. Under the same pigment concentration treatment, the Ci concentration between the two groups showed significant differences (*P* < 0. 05) (Figure 5D). In general, the combination of photosynthetic indices reveals the inhibition of *F. chlamydosporum* on alfalfa photosynthesis in the presence of Cp2 pink pigment.

### Effect of *F. chlamydosporum* on the physiological indexes of alfalfa under different Cp2 pink pigment

**Figure 6:**
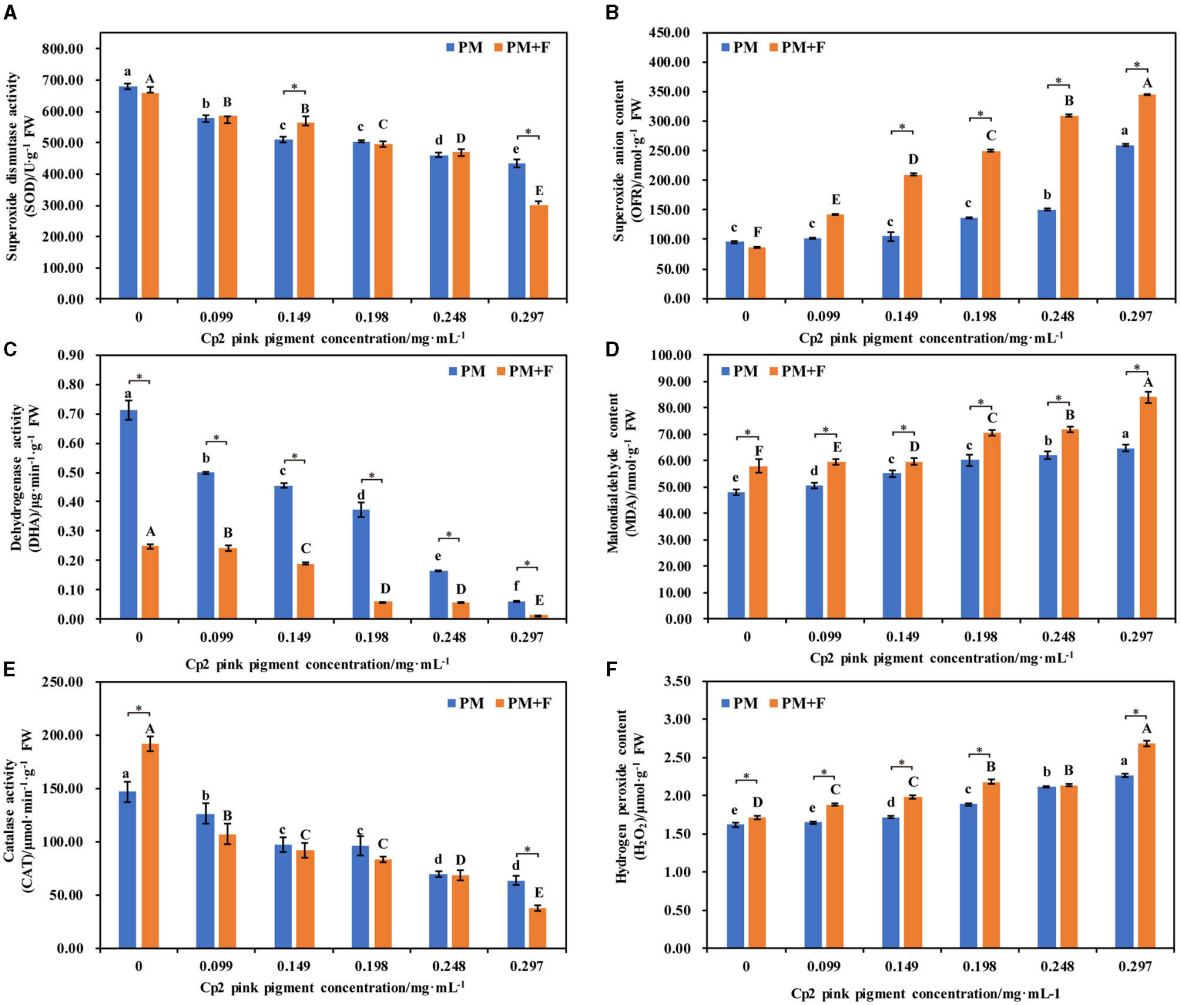
Effects of different concentrations of Cp2 pink pigment and *F. chlamydosporum* on the physiological indexes of alfalfa. **(A)** Superoxide Dismutase activity (SOD). **(B)** Superoxide anion content (OFR). **(C)** Dehydrogenase activity (DHA). **(D)** Malondialdehyde content (MDA). **(E)** Catalase activity (CAT). **(F)** Hydrogen peroxide content (H_2_O_2_). *P < 0.05, (*t*-test, *n* = 8); no * indicate no significant difference (*P* > 0.05).

After 21 days of growth, six physiological indicators of alfalfa were measured under the PM and PM + F groups. The antioxidant enzyme system activity changes, including SOD, DHA, and CAT, were examined first. Compared with the control group, the activities of SOD, DHA, and CAT in the PM and PM + F groups decreased, and this decrease continued as the concentration of Cp2 pink pigment increased. Among them, when the Cp2 pink pigment concentration was 0.297 mg·mL^−1^, SOD, DHA, and CAT activities in the PM and PM + F groups exhibited significant differences (*P* < 0.05) (Figures 6A, C, E). MDA, OFR, and H_2_O_2_ are the products of lipid peroxidation, which indicates the degree of oxidant damage.

In comparison to the control, the levels of MDA, OFR, and H_2_O_2_ in alfalfa in the PM and PM + F groups experienced a gradual increase, while the levels of MDA, OFR, and H_2_O_2_ in alfalfa in the PM + F group remained higher than those in the PM group (Figures 6B, D, F). In addition, when the Cp2 pink pigment concentration was 0.297 mg·mL^−1^, MDA, OFR, and H_2_O_2_ contents in the PM and PM + F groups showed significant differences (*P* < 0.05) compared with the control group. Hence, as compared with CK, the antioxidant enzyme activity in the PM and PM + F groups decreased steadily, while the oxidative damage and lipid peroxidation of the cell membrane continued to increase steadily. Furthermore, the intensity and degree of lipid peroxidation in the PM + F group were greater than those in the PM group, suggesting a more significant inhibitory effect on antioxidant enzyme activity.

### Correlation analysis

**Figure 7:**
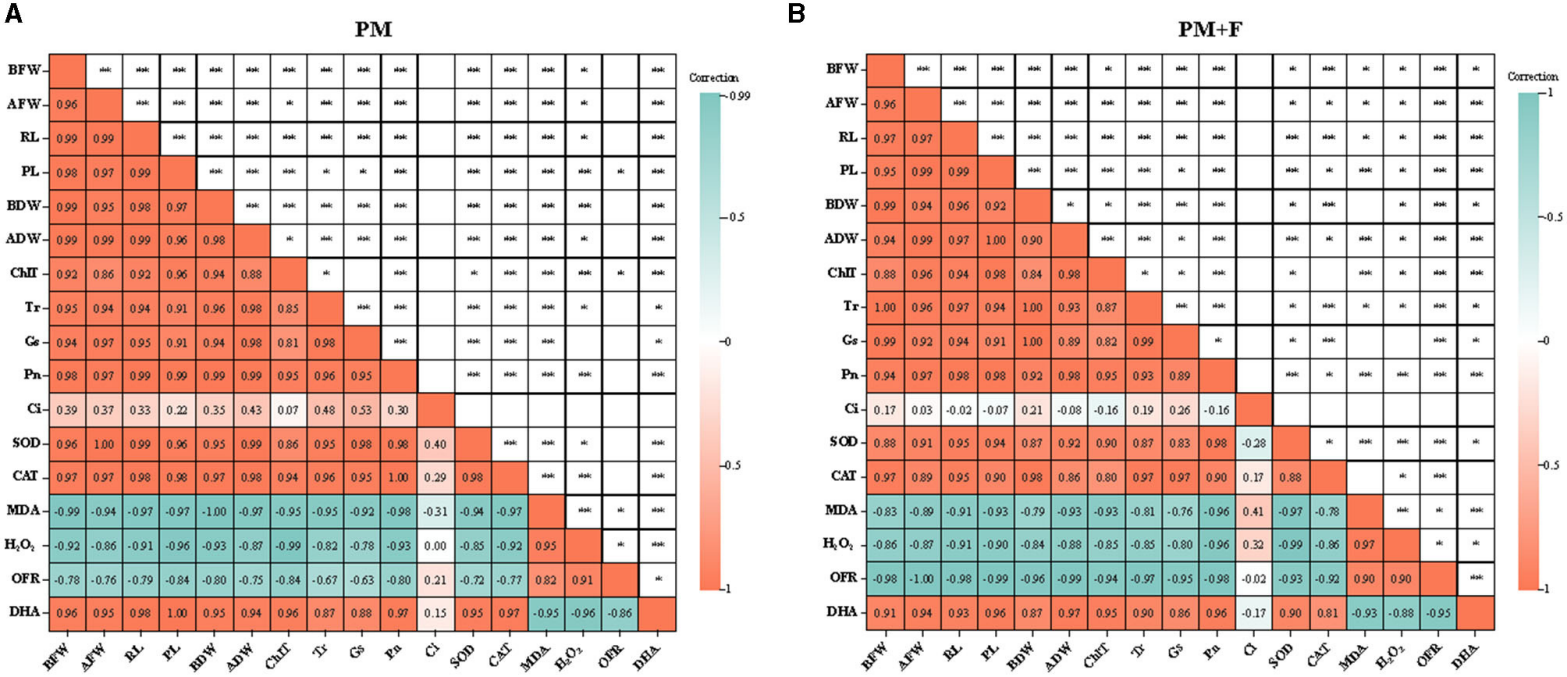
Heatmap of correlation between growth indicators and photosynthetic physiology indicators. The image shows Spearman correlation, and the values in the grid represent correlation coefficients (*r-*values). The red color indicates a positive (0 < r < 1.0) correlation, and the green color indicates a negative (−1.0 < r < 0) correlation. BFW, below-ground fresh weight; AFW, aboveground fresh weight; RL, root length; PL, plant length; BDW, below-ground dry weight; ADW, aboveground dry weight; ChlT, chlorophyll content; Tr, transpiration rate; Gs, stomatal conductance; Pn, photosynthesis rate; Ci, intercellular CO_2_; SOD, superoxide dismutase activity; CAT, catalase activity; MDA, malondialdehyde content; H_2_O_2_, hydrogen peroxide content; OFR, superoxide anion content; and DHA, dehydrogenase activity.

To explore the relationship between plant growth and photosynthesis, the correlation among growth indicators, photosynthetic physiology indicators, and physiological indexes was analyzed using Spearman's correlation analysis. The results revealed significant positive correlations between SOD, CAT, and DHA and FW, AFW, RL, PL, BDW, ADW, ChlT, Tr, Gs, and Pn, and a significant negative correlation between MDA, H_2_O_2_, and OFR in the PM + F group. This suggests a close correlation between changes in FW, AFW, RL, PL, BDW, ADW, ChlT, Tr, Gs, and Pn, and alterations in SOD and CAT. The same was observed for the PM group (Figures 7A, B). Furthermore, Ci was significantly negatively correlated with BFW, BDW, Tr, and Gs but significantly negatively correlated with AFW, RL, PL, ADW, and ChlT in the PM+F group. The results showed a close relationship between Ci and the growth of the aboveground part of alfalfa, PL, and changes in ChlT content. In the PM group, Ci showed a significant negative correlation with MDA and H_2_O_2_, while it showed a positive correlation with all other indicators.

### Principal component analysis

**Figure 8:**
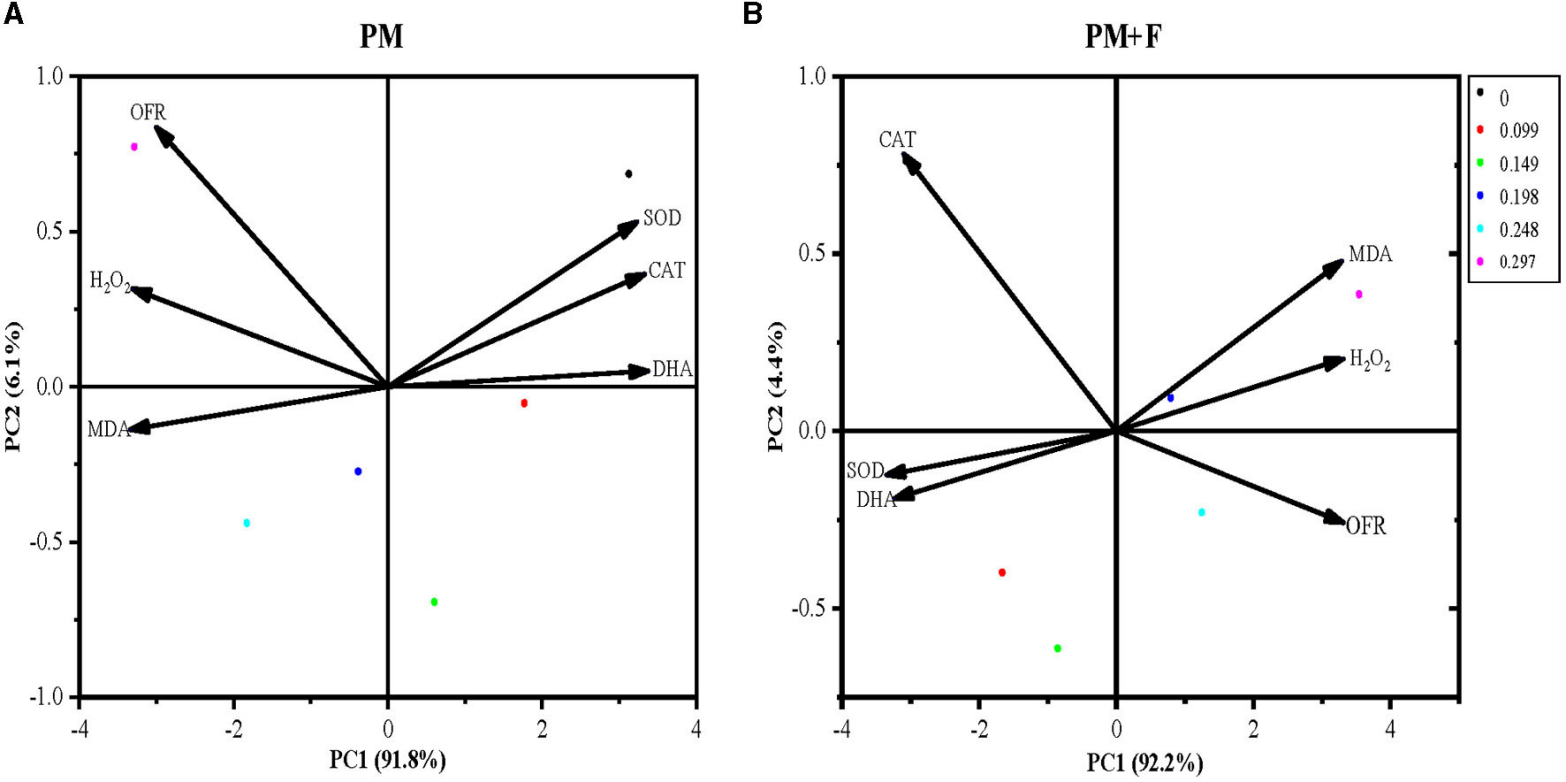
Principal component analysis of physiological indicators. The percent total variance is shown for PC1 and PC2 in parenthesis on the axis. PCA, principal component analysis; PC1, principal component 1; PC2, principal component 2. SOD, superoxide dismutase activity; CAT, catalase activity; MDA, malondialdehyde content; H_2_O_2_, hydrogen peroxide content; OFR, superoxide anion content; and DHA, dehydrogenase activity.

To further clarify the physiological indicators that respond most significantly to the PM and PM + F groups, six indicators for each group were conducted in PCA. The first principal component (PC1) in the PM group explained 91.8%, respectively, of the total variance. While PM + F explained 92.2%. The PM and PM + F groups were well separated on PC1. Therefore, the difference in physiological indicators between the PM and PM + F groups can be explained by PC1 (Figures 8A, B). Next, the PM group treatment with 0.198 mg·mL^−1^, 0.248 mg·mL^−1^, and 0.297 mg·mL^−1^ Cp2 pink pigment showed a positive correlation with alterations in antioxidant enzymes such as SOD, CAT, and DHA and a negative correlation with OFR, MDA, and H_2_O_2_. It is the same as the PM + F group.

## Discussion

### Effect of Cp2 pink pigment on the growth and physiology of alfalfa

During plant infection, pathogenic bacteria secrete and release virulence factors, which play a decisive role during the infection process (Gellatly and Hancock, 2013; Newman et al., 2017). The primary bacterial virulence factors include quorum sensing, biofilm, motility, toxins, and pigment (Poole, 2011; Silva et al., 2016). Bacterial pigments with distinctive chemical structures, serving as virulence factors for pathogenic bacteria, actively contribute to the onset of host diseases by subverting clearance by the host immune response or demonstrating the capability to stimulate an inflammatory response or cellular toxicity (Liu and Nizet, 2009). As reported by Prasannath (2013), plant pathogenic bacteria secrete a large number of virulence factors to disturb the host cells, and bacteria can utilize these virulence factors to bypass, overcome, or suppress the defensive measures of resistant microorganisms, leading to typical symptoms of bacterial diseases such as leaf and fruit spotting, ulceration, vascular wilt, and decay in plants. In this experiment, alfalfas were treated with different concentrations of Cp2 pink pigment for 21 days (PM group), and growth and physiological indicator assays were performed to evaluate alfalfa growth. The results showed that the ChlT content and photosynthetic physiological indicators of alfalfa were impeded as the concentration of pink pigment increased, and the leaves exhibited the typical symptoms of bacterial disease, including leaf chlorosis and withering. The results are similar to the inhibitory symptoms of the toxin secreted by *Pseudomonas syringae* (Arrebola et al., 2011).

Meanwhile, compared with the control group, the suppression of the Cp2 pink pigment on the root system of alfalfa resulted in a more significant decrease in root length, fresh weight, dry weight, total root area, total root volume, average root diameter, and number of roots. This indicates that Cp2 pink pigment plays a role as a virulence factor for *E. persicin*a. Furthermore, in the study, the effects of the Cp2 pink pigment on the antioxidant defense system and lipid peroxidation of alfalfa were evaluated. The activities of SOD, DHA, and CAT in the PM group all exhibited a decreasing trend as the pigment concentration increased. MDA, OFR, and H_2_O_2_ content showed an overall increasing and then decreasing trend with increasing Cp2 pink pigment concentration. This is consistent with the inhibitory effect of pyocyanin on algal physiology (Lau et al., 2004). According to the findings, Cp2 pink pigment can induce a surge of ROS in alfalfa despite the significant reduction in antioxidant enzyme activity. The O${}_{2}^{-}$and H_2_O_2_ generated within the alfalfa are unable to be eliminated promptly, leading to oxidative damage in the alfalfa and a substantial rise in the levels of lipid peroxidation products such as MDA, OFR, and H_2_O_2_. This series of biochemical reactions is initiated by the complex sensory system of plants upon perception of pathogen chemical signals (De Gara et al., 2003). ROS are involved in plant metabolic activity, but their excessive production under stress can trigger ROS bursts in alfalfa (Raja et al., 2017). The excessive accumulation of ROS can result in photooxidative damage to various solid cells, such as carbohydrates, lipids, nucleic acids, and proteins (Mansoor et al., 2022). This can cause oxidative stress in plant cells and ultimately lead to death (Mansoor et al., 2022). Meanwhile, a significant surge in ROS can place a heavy load on the antioxidant defense system (Lamb and Dixon, 1997), potentially leading to a decline in the antioxidant enzyme activity of alfalfa. The growth of alfalfa was evaluated by measuring growth and physiological indicators. It was discovered that the pathogenic effect of Cp2 pink pigment on alfalfa was similar to the disease symptoms triggered by plant pathogens. This indicates that Cp2 pink pigment is one of the potential virulence factors secreted by pathogenic bacteria that could exacerbate the infection process of the pathogenic bacteria on alfalfa, thereby supporting its survival and proliferation (Ni et al., 2020).

### Effect of Cp2 pink pigment and *F. chlamydosporum* on the growth and physiology of alfalfa

*Fusarium* is a well-known plant pathogenic fungus that causes diseases in a wide range of plants, including fruit crops, vegetables, cereal grains, and ornamentals (Summerell et al., 2003; Michielse and Rep, 2009; Tembhurne et al., 2017; Yang et al., 2022; Buttar et al., 2023). *F. chlamydosporum* is a soil-borne pathogen that is widely distributed, has the ability to survive for extended periods in soil, and can act as a facultative parasite, infecting alfalfa (Cao et al., 2022). In severe cases, *F. chlamydosporum* can cause the death of the entire plant by disrupting vital processes such as material absorption and transport, photosynthesis, and material synthesis and transformation, which can inhibit the normal growth of alfalfa. The results of this experiment can also confirm this point, as stated in the study.

The study found that the growth indicators, physiological indicators, and photosynthetic physiological indexes of alfalfa were reduced after the addition of fungal suspension compared with the control. Furthermore, alfalfa is affected by the combined effects of soil-borne pathogenic fungi and rhizosphere microbial metabolites during its growth and development. What was the role of rhizosphere microbial metabolites in this process? In the study, compared with the PM group, the growth of alfalfa was more severely inhibited with the addition of varying concentrations of Cp2 pink pigment. The potential virulence factor of Cp2, which is secreted by the pigment, interacts with *F. chlamydosporum* and causes the infection of alfalfa as a new symbiotic agent (Adnani et al., 2017). This, in turn, may be due to the fact that pigment is one of the factors that contribute to virulence. Furthermore, the results of the in-dish antagonistic effect of Cp2 pink pigment on *F. chlamydosporum* showed that the growth inhibition rate of Cp2 pink pigment on the pathogen reached more than 40% in the first 6 days. However, after 6 days of inoculation, Cp2 pink pigment had no inhibitory effect on its growth (Zhang et al., 2022). In this experiment, the growth indicators of alfalfa seedlings were measured 21 days after inoculation, and the results may be attributed to the potent combination of Cp2 pink pigment and *F. chlamydosporum*, which collaborate to combat each other. Furthermore, this could pertain to the indole compound usambarensine, which is a component of Cp2 pink pigment (Zhang et al., 2022). Another possibility is that usambarensine acts as an indole compound, serving as an intracellular signaling molecule that facilitates the colonization of *F. chlamydosporum* in alfalfa (Kim and Park, 2015). This also suggests the interaction between the Cp2 pink pigment present in plants and soil-borne pathogenic fungi. This also raises the possibility that Cp2 pink pigment could be used as a plant immunomodulator agent in model plants.

## Data availability statement

The raw data supporting the conclusions of this article will be made available by the authors, without undue reservation.

## Author contributions

RH: Writing—original draft, Writing—review and editing. HZ: Writing—review and editing. HC: Writing—review and editing. LH: Writing—review and editing. XL: Funding acquisition, Writing—review and editing. ZZ: Writing—review and editing.
